# Stress-induced rupioid psoriasis managed with ustekinumab

**DOI:** 10.1093/skinhd/vzag018

**Published:** 2026-03-25

**Authors:** Gustavo Almeida-Silva, Joana Antunes

**Affiliations:** Dermatology Department, Unidade Local de Saúde Santa Maria, Lisbon, Portugal; Dermatology University Clinic, Faculdade de Medicina da Universidade de Lisboa, Lisbon, Portugal; Dermatology Department, Unidade Local de Saúde Santa Maria, Lisbon, Portugal; Dermatology University Clinic, Faculdade de Medicina da Universidade de Lisboa, Lisbon, Portugal

## Abstract

We report the first case of stress-induced rupioid psoriasis, a rare and severe hyperkeratotic variant of psoriasis, arising after the sudden loss of a close relative. The patient achieved remarkable clearance with ustekinumab, highlighting the potential of interleukin (IL)-12/IL-23 blockade in this under-recognized phenotype. This case underscores the role of acute stress as a trigger, and the urgent need to better define the pathophysiology and tailored treatment of hyperkeratotic psoriasis variants.

Dear Editor, A 22-year-old man presented to our department with a 3-month history of limpet-like hyperkeratotic erythematous plaques all over his body, which had developed following the death of his mother. Physical examination showed widespread infiltrated, erythematous and well-demarcated arciform plaques with a thick and adherent hyperkeratotic silver scale (more evident along the borders) that affected his entire torso, where plaques were confluent, forming geometrical shapes (some with central clearing, with diameters ranging up to 30 cm), and the upper and lower limbs (anterior and posterior surfaces). Some of the lesions had cone-shaped scales, which granted them a limpet-like appearance, consistent with rupioid psoriasis. The dermatosis affected approximately 60% of the patient’s body surface area (BSA). There was neither nail involvement nor joint complaints. His baseline Psoriasis Area and Severity Index (PASI) and Dermatology Life Quality Index (DLQI) scores were 26.8 and 17, respectively (Figure 1).

**Figure 1:**
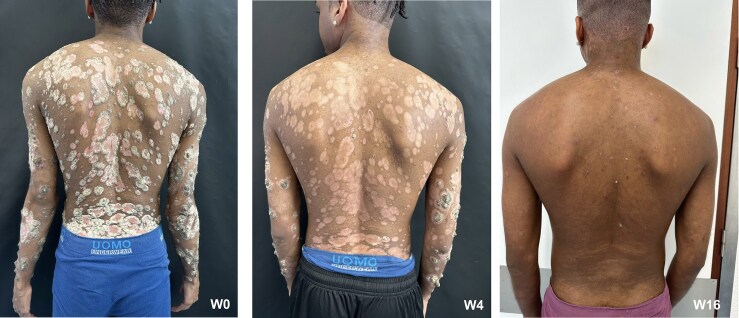
Clinical presentation of the patient. Left: thick hyperkeratotic limpet-like plaques are seen on the patient’s back at baseline (week 0). Middle: after 4 weeks of ustekinumab treatment, marked improvement is seen. Right: after 16 weeks of ustekinumab treatment, showing significantly fewer lesions and marked improvement.

The patient’s medical history included plaque psoriasis (diagnosed and histologically confirmed in 2014), which had been in remission on adalimumab 40 mg every 2 weeks for several years, until he underwent this traumatic event. The patient was switched to ustekinumab 45 mg, which cleared most of the psoriatic lesions, leaving only postinflammatory hypopigmentation. At week 24, his PASI and DLQI scores were 3.2 and 0, respectively, with only minimal persistent plaques on his torso (BSA 3%).

There are three well-described variants of hyperkeratotic psoriasis: elephantine, ostraceous and rupioid (limpet-like).^1^ Rupioid psoriasis is a rare and severe variant of psoriasis, characterized by thick, conical, crusted plaques resembling limpet shells. These hyperkeratotic lesions often indicate poorly controlled or treatment-resistant disease.^2^ This variant has also been linked with immunosuppression, particularly HIV infection, prompting screening in all patients.^3^ To our knowledge, only 20 cases of rupioid psoriasis have been reported in the literature, indicating that this variant is probably under-reported.^1^

We present the first case report of rupioid psoriasis arising after acute stress, a known trigger for psoriasis flares.^4^ To date, unlike other variants (such as generalized pustular psoriasis), no specific pathophysiological pathway has been outlined for hyperkeratotic psoriasis variants, meaning a tailored approach is not yet possible.

## Data Availability

The data underlying this article will be shared on reasonable request to the corresponding author.
